# Supplementary material for: Association of Genes TRH, PRL and PRLR with Milk Performance, Reproductive Traits and Heat Stress Response in Dairy Cattle
Source: Int J Mol Sci. 2025 Feb 24;26(5):1963. doi: 10.3390/ijms26051963 (PMC11901056; doi:10.3390/ijms26051963)
Supplement: Supplementary file 1 [file ijms-26-01963-s001.zip › IJMS-Supplementary Materials.pdf]

Association of Gene *TRH*, *PRL* and *PRLR* with Milk Performance, Reproductive Traits and Heat Stress Response in Dairy Cattle

1 Supplementary Methods

1. Genetic evaluation for milk performance traits

Milk performance traits included milk yield (MY), protein yield (PY), fat yield (FY), and somatic cell score (SCS). The phenotypes for above traits were from test-day DHI records. Test-day FY and PY are calculated from test-day MY and fat percentage (FP), and protein percentage (PP), respectively. SCS is derived from somatic cell count (SCC) through a logarithmic transformation.

For the genetic evaluation of four milk performance traits (MY, PY, FY and SCS), a single-trait random regression test-day model was used to obtain the estimated breeding values (EBVs) for different lactations, respectively. The submodel used to fit the regression curve was a fourth-order Legendre polynomial. The genetic evaluation was performed using the DMU software. The fixed effects included farm-test day and calving year-season.

2. Genetic evaluation for reproduction traits

Reproductive traits included age at the first service in heifers (AFS), age at the first calving in heifers (AFC), conception rate (CR; 1 = pregnant, 0 = non-pregnant) at first insemination, the interval from the first to last insemination in heifers (IFL_H) and cows (IFL_C), stillbirth in heifers (SB_H) and cows (SB_C) (1 = calf was alive 24 h after birth and 2 = calf was dead), and calving ease in heifers (CE_H) and cows (CE_C). And the scores of CE from 1 to 3, in which 1 = unassisted, 2 = easy pull, 3 = hard pull and surgery needed.

Genetic evaluations for AFS, AFC, IFL_H, SB_H, and CE_H were performed using an animal model, while a repeatability animal model was applied to CR, IFL_C, SB_C, and CE_C. The evaluations were carried out using the Average Information (AI)-REML procedure, as implemented in the DMU package. Furthermore, the evaluation model included several fixed effects, such as the fixed effects of the birth farm-year, the birth year-month, insemination technician, the first insemination farm and year within lactation, the first insemination year and month within lactation, gender-controlled semen of the first insemination, age at first service, the calving farm and year, the calving year and month, calf sex, and parity. The specific effects included in the models varied depending on the trait being evaluated. More information about IFL and CR can be found in study by Liu et al. (2017); SB and CE can be found in study by Chen et al. (2021); and AFS and AFC can be found in study by Zhu et al. (2024).

3. Genetic evaluation for heat stress response traits

The heat stress response traits included rectal temperature (RT), respiratory rate (RR), and drooling score (DS).

Rectal temperature was measured using a digital thermometer (Omron MC-347, Omron

Corp., Osaka, Japan). The RR and DS measurement protocols can be described as follows. RR score 1: respiration rate is normal, and it is difficult to visualize the movement of chest and rumen; RR score 2: respiration rate is in the range of 80 to 120 times/min and small visual signs of movement of chest and rumen are observed; and RR score 3: respiration rate is greater than 120 times/min, and there are clear signs of the movement of chest and rumen with joggling of body and open-mouthed respiration. DS score 1: no drooling and normal condition of jaw; DS score 2: some drooling and some filament saliva flow out of the mouth and a wet jaw may be observed; and DS score 3: excessive drooling and large amount of saliva flowing out of the mouth and wet jaw.

Genetic evaluation was performed based on a repeatability animal model, using the average information (AI)-REML procedure implemented in the DMU package. The systematic effects in evaluation model included farm-year for RT or farm-year-scoring-person for RR and DS, parity, lactation stage, milking status and THI. More information can be found in study by Luo et al. (2021).

2 **Supplementary Materials**

Figure S1. The relative mRNA expression of *PRLR* in Holstein cows with alleles AA, AC and CC of g.39099173C/A.

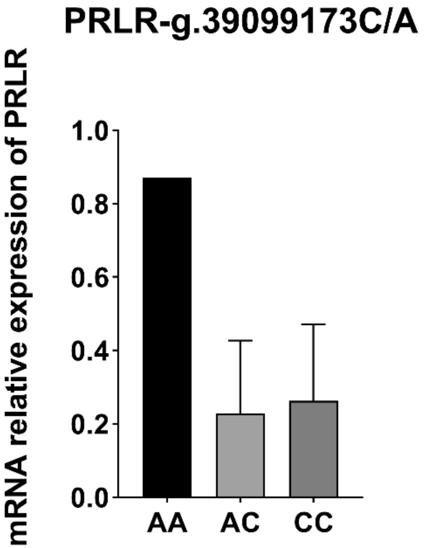

Figure S2. Distribution of estimated breeding values for 24 traits in Holstein population

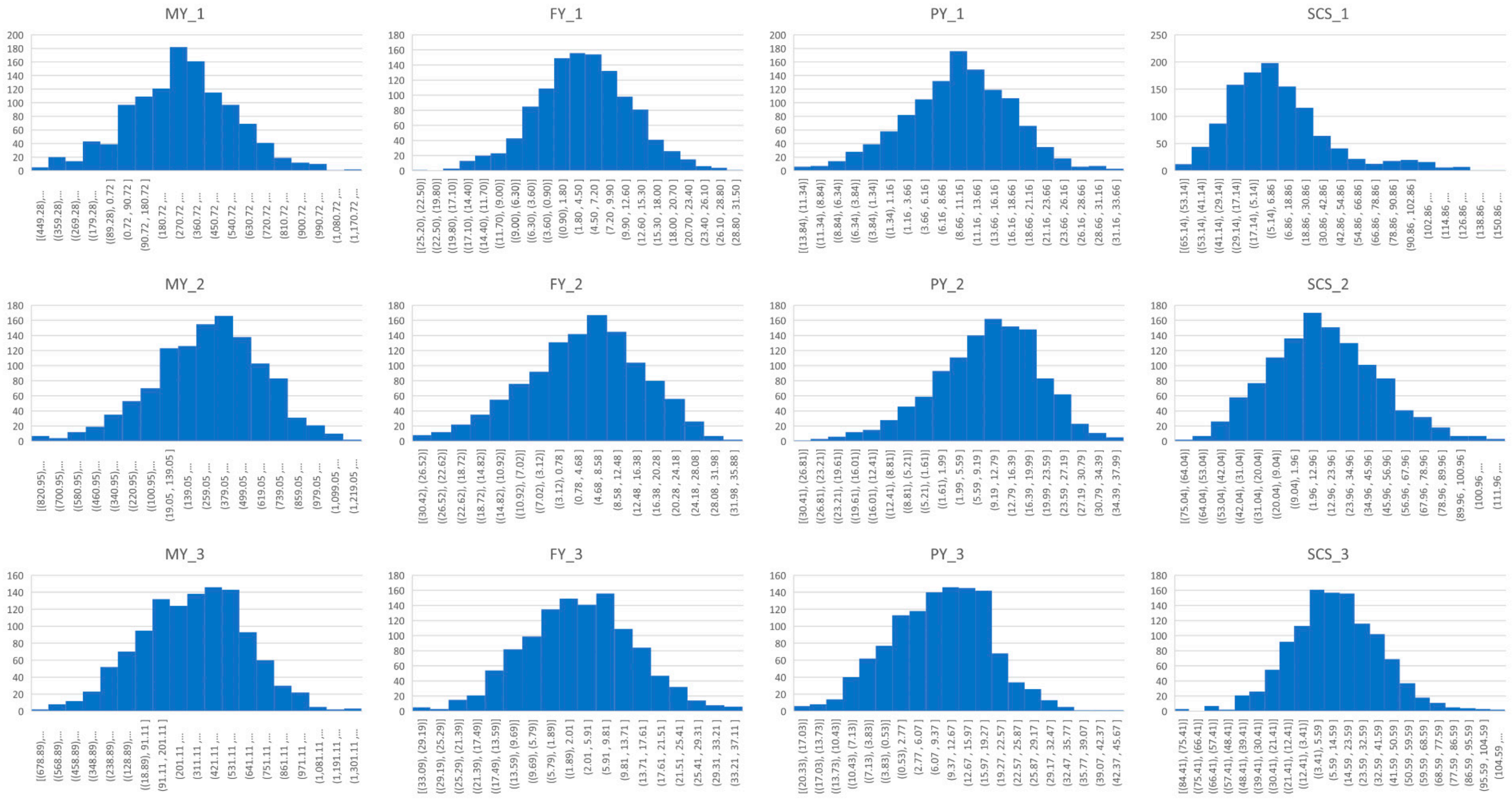

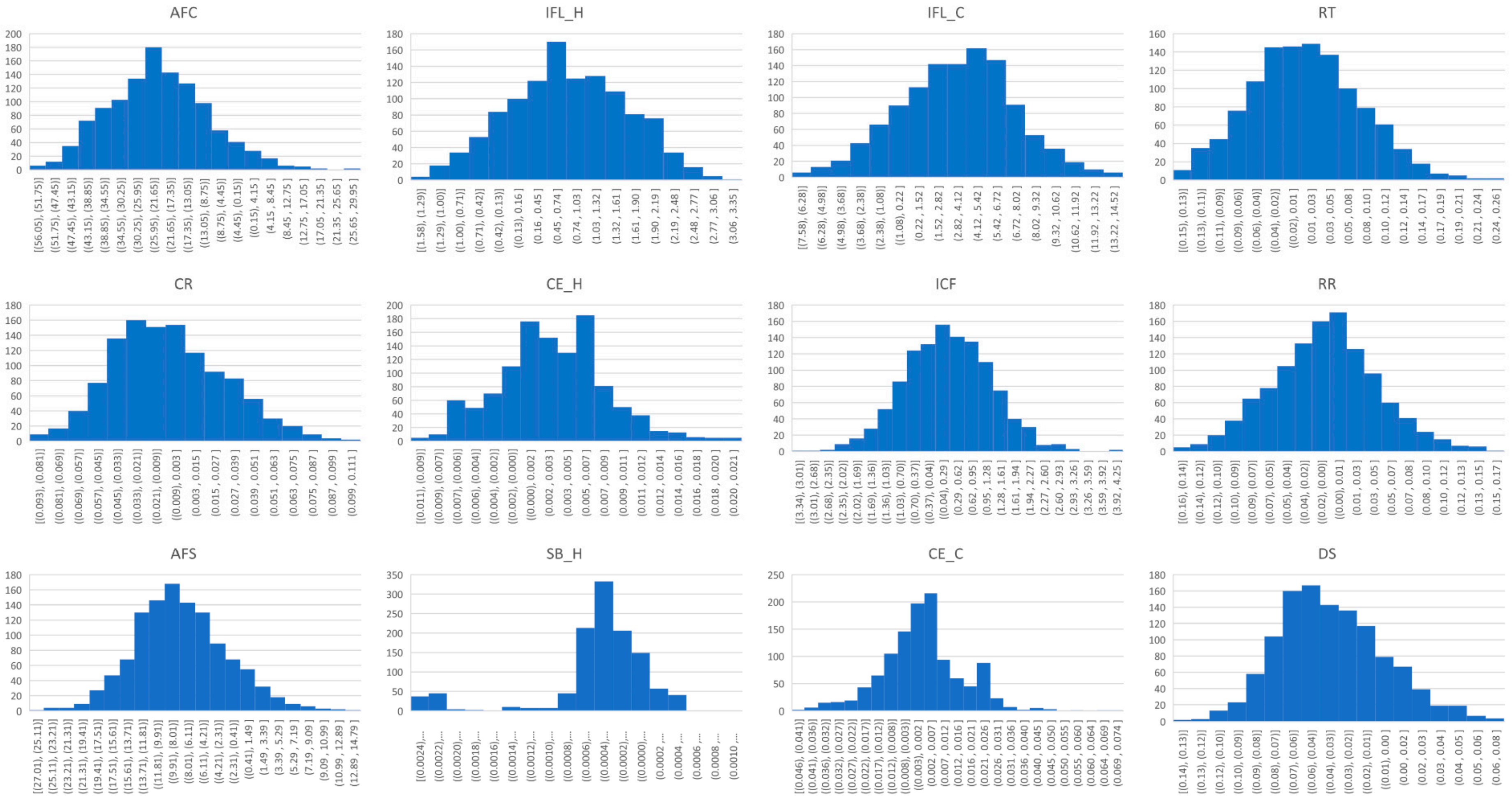
AFC
[(56.05), (51.75)]
((51.75), (47.45)]
((47.45), (43.15)]
((43.15), (38.85)]
((38.85), (34.55)]
((34.55), (30.25)]
((30.25), (25.95)]
((25.95), (21.65)]
((21.65), (17.35)]
((17.35), (13.05)]
((13.05), (8.75)]
((8.75), (4.45)]
((4.45), (0.15)]
((0.15), 4.15]
(4.15 , 8.45]
(8.45 , 12.75]
(12.75 , 17.05]
(17.05 , 21.35]
(21.35 , 25.65]
(25.65 , 29.95]
IFL_H
[(1.58), (1.29)]
((1.29), (1.00)]
((1.00), (0.71)]
((0.71), (0.42)]
((0.42), (0.13)]
((0.13), 0.16]
(0.16 , 0.45]
(0.45 , 0.74]
(0.74 , 1.03]
(1.03 , 1.32]
(1.32 , 1.61]
(1.61 , 1.90]
(1.90 , 2.19]
(2.19 , 2.48]
(2.48 , 2.77]
(2.77 , 3.06]
(3.06 , 3.35]
IFL_C
[(7.58), (6.28)]
((6.28), (4.98)]
((4.98), (3.68)]
((3.68), (2.38)]
((2.38), (1.08)]
((1.08), 0.22]
(0.22 , 1.52]
(1.52 , 2.82]
(2.82 , 4.12]
(4.12 , 5.42]
(5.42 , 6.72]
(6.72 , 8.02]
(8.02 , 9.32]
(9.32 , 10.62]
(10.62 , 11.92]
(11.92 , 13.22]
(13.22 , 14.52]
RT
[(0.15), (0.13)]
((0.13), (0.11)]
((0.11), (0.09)]
((0.09), (0.06)]
((0.06), (0.04)]
((0.04), (0.02)]
((0.02), 0.01]
(0.01 , 0.03]
(0.03 , 0.05]
(0.05 , 0.08]
(0.08 , 0.10]
(0.10 , 0.12]
(0.12 , 0.14]
(0.14 , 0.17]
(0.17 , 0.19]
(0.19 , 0.21]
(0.21 , 0.24]
(0.24 , 0.26]
CR
[(0.093), (0.081)]
((0.081), (0.069)]
((0.069), (0.057)]
((0.057), (0.045)]
((0.045), (0.033)]
((0.033), (0.021)]
((0.021), (0.009)]
((0.009), 0.003]
(0.003 , 0.015]
(0.015 , 0.027]
(0.027 , 0.039]
(0.039 , 0.051]
(0.051 , 0.063]
(0.063 , 0.075]
(0.075 , 0.087]
(0.087 , 0.099]
(0.099 , 0.111]
CE_H
[(0.011), (0.009)]
((0.009), (0.007)]
((0.007), (0.006)]
((0.006), (0.004)]
((0.004), (0.002)]
((0.002), (0.000)]
((0.000), 0.002]
(0.002 , 0.003]
(0.003 , 0.005]
(0.005 , 0.007]
(0.007 , 0.009]
(0.009 , 0.011]
(0.011 , 0.012]
(0.012 , 0.014]
(0.014 , 0.016]
(0.016 , 0.018]
(0.018 , 0.020]
(0.020 , 0.021]
ICF
[(3.34), (3.01)]
((3.01), (2.68)]
((2.68), (2.35)]
((2.35), (2.02)]
((2.02), (1.69)]
((1.69), (1.36)]
((1.36), (1.03)]
((1.03), (0.70)]
((0.70), (0.37)]
((0.37), (0.04)]
((0.04), 0.29]
(0.29 , 0.62]
(0.62 , 0.95]
(0.95 , 1.28]
(1.28 , 1.61]
(1.61 , 1.94]
(1.94 , 2.27]
(2.27 , 2.60]
(2.60 , 2.93]
(2.93 , 3.26]
(3.26 , 3.59]
(3.59 , 3.92]
(3.92 , 4.25]
RR
[(0.16), (0.14)]
((0.14), (0.12)]
((0.12), (0.10)]
((0.10), (0.09)]
((0.09), (0.07)]
((0.07), (0.05)]
((0.05), (0.04)]
((0.04), (0.02)]
((0.02), (0.00)]
((0.00), 0.01]
(0.01 , 0.03]
(0.03 , 0.05]
(0.05 , 0.07]
(0.07 , 0.08]
(0.08 , 0.10]
(0.10 , 0.12]
(0.12 , 0.13]
(0.13 , 0.15]
(0.15 , 0.17]
AFS
[(27.01), (25.11)]
((25.11), (23.21)]
((23.21), (21.31)]
((21.31), (19.41)]
((19.41), (17.51)]
((17.51), (15.61)]
((15.61), (13.71)]
((13.71), (11.81)]
((11.81), (9.91)]
((9.91), (8.01)]
((8.01), (6.11)]
((6.11), (4.21)]
((4.21), (2.31)]
((2.31), (0.41)]
((0.41), 1.49]
(1.49 , 3.39]
(3.39 , 5.29]
(5.29 , 7.19]
(7.19 , 9.09]
(9.09 , 10.99]
(10.99 , 12.89]
(12.89 , 14.79]
SB_H
[(0.0024),...
((0.0022),...
((0.0020),...
((0.0018),...
((0.0016),...
((0.0014),...
((0.0012),...
((0.0010),...
((0.0008),...
((0.0006),...
((0.0004),...
((0.0002),...
((0.0000),...
(0.0002 ,...
(0.0004 ,...
(0.0006 ,...
(0.0008 ,...
(0.0010 ,...
CE_C
[(0.046), (0.041)]
((0.041), (0.036)]
((0.036), (0.032)]
((0.032), (0.027)]
((0.027), (0.022)]
((0.022), (0.017)]
((0.017), (0.012)]
((0.012), (0.008)]
((0.008), (0.003)]
((0.003), 0.002]
(0.002 , 0.007]
(0.007 , 0.012]
(0.012 , 0.016]
(0.016 , 0.021]
(0.021 , 0.026]
(0.026 , 0.031]
(0.031 , 0.036]
(0.036 , 0.040]
(0.040 , 0.045]
(0.045 , 0.050]
(0.050 , 0.055]
(0.055 , 0.060]
(0.060 , 0.064]
(0.064 , 0.069]
(0.069 , 0.074]
DS
[(0.14), (0.13)]
((0.13), (0.12)]
((0.12), (0.10)]
((0.10), (0.09)]
((0.09), (0.08)]
((0.08), (0.07)]
((0.07), (0.06)]
((0.06), (0.04)]
((0.04), (0.03)]
((0.03), (0.02)]
((0.02), (0.01)]
((0.01), 0.00]
(0.00 , 0.02]
(0.02 , 0.03]
(0.03 , 0.04]
(0.04 , 0.05]
(0.05 , 0.06]
(0.06 , 0.08]

*Milk yield (MY), fat yield (FY), protein yield (PY), and somatic cells score (SCS), and age at the first service (AFS) and calving (AFC), and conception rate of first insemination (CR), the interval from the first to last insemination in heifers (IFL_H) and cows (IFL_C), stillbirth in heifers (SB_H) and cows (SB_C), and calving ease in heifers (CE_H) and cows (CE_C).

References

1 Liu, A.; Lund, M.S.; Wang, Y.; Guo, G.; Dong, G.; Madsen, P.; Su, G. Variance components and correlations of female fertility traits in Chinese Holstein population. *J Anim Sci Biotechnol*. 2017, *8*, 56. doi: 10.1186/s40104-017-0189-x.

2 Chen, Z.; Brito, L.F.; Luo, H.; Shi, R.; Chang, Y.; Liu, L.; Guo, G.; Wang, Y. Genetic and Genomic Analyses of Service Sire Effect on Female Reproductive Traits in Holstein Cattle. *Front Genet*. 2021, *12*, 713575. doi: 10.3389/fgene.2021.713575.

3 Zhu, K.; Li, T.; Liu, D.; Wang, S.; Wang, S.; Wang, Q.; Pan, Y.; Zan, L.; Ma, P. Estimation of genetic parameters for fertility traits in Chinese Holstein of south China. *Front Genet*. 2024, *14*, 1288375. doi: 10.3389/fgene.2023.1288375.

4 Luo, H.; Brito, L.F.; Li, X.; Su, G.; Dou, J.; Xu, W.; Yan, X.; Zhang, H.; Guo, G.; Liu, L.; Wang, Y. Genetic parameters for rectal temperature, respiration rate, and drooling score in Holstein cattle and their relationships with various fertility, production, body conformation, and health traits. *J Dairy Sci*. 2021, *104*, 4390-4403. doi: 10.3168/jds.2020-19192.
